# Characterization of Altered Molecular Pathways in the Entorhinal Cortex of Alzheimer’s Disease Patients and In Silico Prediction of Potential Repurposable Drugs

**DOI:** 10.3390/genes13040703

**Published:** 2022-04-15

**Authors:** Paolo Fagone, Katia Mangano, Gabriella Martino, Maria Catena Quattropani, Manuela Pennisi, Rita Bella, Francesco Fisicaro, Ferdinando Nicoletti, Maria Cristina Petralia

**Affiliations:** 1 Department of Biomedical and Biotechnological Sciences, University of Catania, Via S. Sofia 89, 95123 Catania, Italy; paolofagone@yahoo.it (P.F.); kmangano@unict.it (K.M.); manuela.pennisi@unict.it (M.P.); 2 Department of Clinical and Experimental Medicine, University of Messina, 98122 Messina, Italy; gabriella.martino@unime.it (G.M.); m.cristinapetralia@gmail.com (M.C.P.); 3 Department of Educational Sciences, University of Catania, Via Teatro Greco 84, 95124 Catania, Italy; maria.quattropani@unict.it; 4 Department of Medical and Surgical Sciences and Advanced Technologies, University of Catania, Via S. Sofia 78, 95123 Catania, Italy; rbella@unict.it (R.B.); drfrancescofisicaro@gmail.com (F.F.)

**Keywords:** Alzheimer’s disease, entorhinal cortex, meta-analysis, in silico pharmacology

## Abstract

Alzheimer’s disease (AD) is the most common cause of dementia worldwide and is characterized by a progressive decline in cognitive functions. Accumulation of amyloid-β plaques and neurofibrillary tangles are a typical feature of AD neuropathological changes. The entorhinal cortex (EC) is the first brain area associated with pathologic changes in AD, even preceding atrophy of the hippocampus. In the current study, we have performed a meta-analysis of publicly available expression data sets of the entorhinal cortex (EC) in order to identify potential pathways underlying AD pathology. The meta-analysis identified 1915 differentially expressed genes (DEGs) between the EC from normal and AD patients. Among the downregulated DEGs, we found a significant enrichment of biological processes pertaining to the “neuronal system” (R-HSA-112316) and the “synaptic signaling” (GO:0099536), while the “regulation of protein catabolic process” (GO:00042176) and “transport of small molecules” (R-HSA-382551) resulted in enrichment among both the upregulated and downregulated DEGs. Finally, by means of an in silico pharmacology approach, we have prioritized drugs and molecules potentially able to revert the transcriptional changes associated with AD pathology. The drugs with a mostly anti-correlated signature were: efavirenz, an anti-retroviral drug; tacrolimus, a calcineurin inhibitor; and sirolimus, an mTOR inhibitor. Among the predicted drugs, those potentially able to cross the blood-brain barrier have also been identified. Overall, our study found a disease-specific set of dysfunctional biological pathways characterizing the EC in AD patients and identified a set of drugs that could in the future be exploited as potential therapeutic strategies. The approach used in the current study has some limitations, as it does not account for possible post-transcriptional events regulating the cellular phenotype, and also, much clinical information about the samples included in the meta-analysis was not available. However, despite these limitations, our study sets the basis for future investigations on the pathogenetic processes occurring in AD and proposes the repurposing of currently used drugs for the treatment of AD patients.

## 1. Introduction

Alzheimer’s disease (AD) is a chronic and progressive neurodegenerative disease that represents the principal cause of age-associated dementia worldwide [1]. Its main features are the amyloid plaques deposition and the tau neurofibrillary tangles formation, which cause loss of synaptic plasticity, thus impairing cognitive functions [2,3,4,5]. Alterations in the homeostasis of glutamate and Ca^2+^ have also been associated with the pathogenesis of AD [6,7]. Various risk factors have been identified so far, including non-modifiable genetic factors (e.g., APO-E4 allele, mutations in PSN-1-2 and APP genes) [8] and modifiable factors (physical activity, diet, obesity, hypertension, diabetes, smoking, alcohol consumption) [9].

Typically, in AD patients, progressive brain atrophy is observed that starts in the parahippocampal cortex and progresses in the hippocampus, the temporal lobe, and the parietal lobe, and finally spreads to the entire cortex in the advanced stages of the disease [10,11]. MRI analysis has revealed that the entorhinal cortex (EC) is the first brain area to be affected in AD, even before the atrophy of the hippocampus occurs [12]. The EC is located in the medial temporal lobe, adjacent to the hippocampus. From a neurophysiological standpoint, the EC plays a critical role in memory consolidation. Reduced EC volume provides the best discrimination between normal subjects and mild cognitive impairment (MCI) or AD patients. The EC provides the hippocampus with the majority of the cortical sensory and associational input; hence, EC degeneration can disrupt hippocampal processing, which is dependent on entorhinal inputs [13]. The superficial layers of the EC receive inputs from cortical regions, while layer II and III EC neurons deliver cortical sensory input to the hippocampus. In particular, the EC layer II stellate neurons provide input to the dentate gyrus (DG) and hippocampal CA3 region, while the layer III EC pyramidal neurons project to the CA1 region [13]. For these reasons, the memory dysfunction observed in AD is believed to be linked to the progressive degeneration of the EC and, consequently, of its targets, represented by the DG and the CA3 and CA1 areas in the hippocampus [13]. The early involvement of EC during the course of AD progression makes this particular brain region an area of profound interest, as targeting the aberrant pathways that affect EC at the initial stages of the disease could be more effective in reducing the progression of the disease.

**Figure 1:**
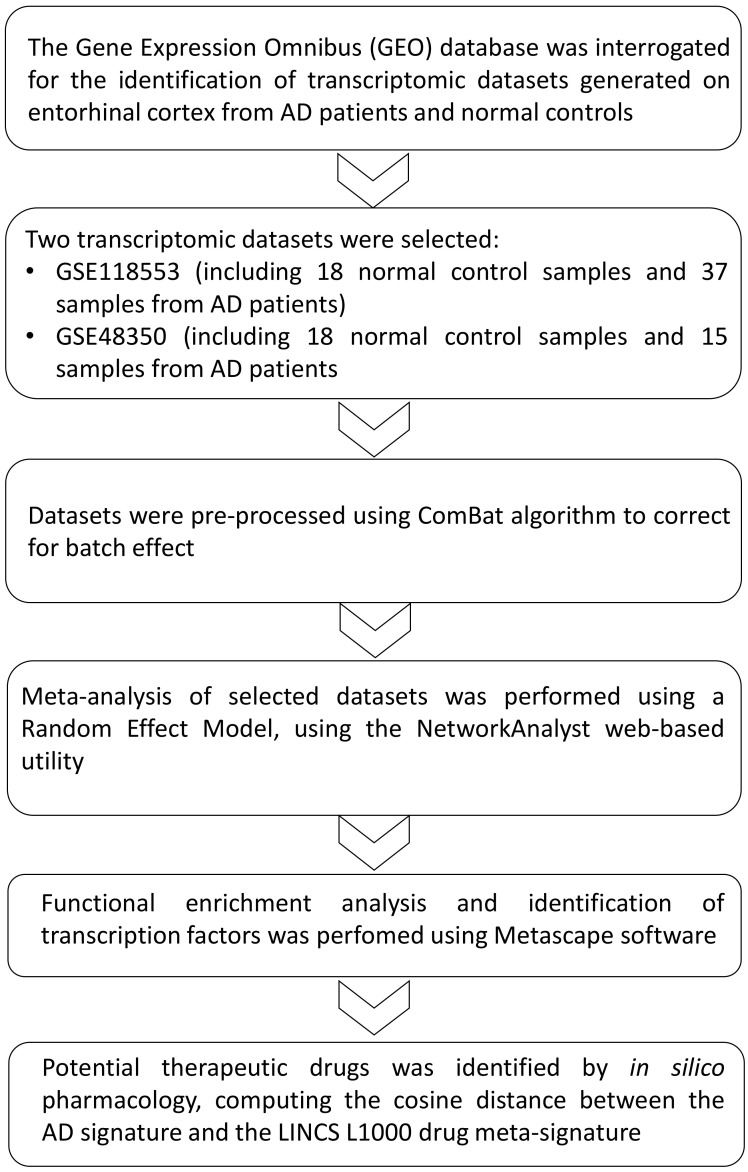
Experimental layout. The GEO database was interrogated to identify transcriptomic data sets generated on the entorhinal cortex of AD patients and normal subjects. Two data sets (GSE118554 and GSE48350) passed the inclusion criteria and were selected for further analysis. After a preprocessing step, the differentially expressed genes (DEGs) were obtained by performing a meta-analysis using a random-effect model. Functional enrichment analysis was performed on the DEGs, and an *in silico* pharmacology approach was employed to predict potential novel therapeutic options.

Several groups have explored the gene expression signatures in AD brain samples in order to better characterize the molecular mechanisms underlying AD pathology; however, the molecular pathways involved in the vulnerability of the EC during AD and target-specific pharmacological treatments are not available [14,15,16,17,18]. Whole-genome transcriptomic data sets have been extensively exploited to decipher pathogenic processes and to identify therapeutic targets in several settings, including neurodegeneration [1], autoimmune diseases [19,20,21], and cancer [22,23]. The meta-analysis of previously published transcriptomic studies aims at increasing the power of the analysis and to better define gene expression differences among selected populations. In the present study, we have performed a meta-analysis of two publicly available expression data sets with the aim to identify molecular pathways underlying AD pathology. Then, we have proposed a number of drugs that may be potentially able to revert the transcriptional changes associated with AD pathology (Figure 1 shows the overall layout of the experimental design). The approach used in the current study has some limitations, as it does not account for possible post-transcriptional events regulating the cellular phenotype, and also, much clinical information about the samples included in the meta-analysis was not available. Moreover, no transcriptional data are currently available on the initial EC aberrations that could be observed in patients suffering from MCI. However, despite these shortcomings, our study sets the basis for future investigations on the pathogenetic processes occurring in AD and proposes the repurposing of currently used drugs for the treatment of AD patients.

## 2. Materials and Methods

### 2.1. Dataset Selection

The NCBI Gene Expression Omnibus (GEO) database (http://www.ncbi.nlm.nih.gov/geo/, accessed on 10 January 2022) was used to identify transcriptomic data sets comparing the expression profiles of entorhinal cortex from control donors and AD patients. The GEO database was manually searched using the MeSH terms (medical subject headings) “Alzheimer’s disease” and “entorhinal cortex”. The collected data sets were further selected if they met the following inclusion criteria: (a) expression profiling; (b) samples from the entorhinal cortex; (c) included one cohort of AD patients and another cohort of age-matched control people; and (d) species of origin “Homo sapiens.” Finally, two data sets were included in the meta-analysis: GSE118553 [17] and GSE48350 [18]. Briefly, the GSE118553 data set included 18 normal control samples and 37 samples from AD patients. The GSE48350 data set included 18 normal control samples and 15 samples from AD patients. The patients and controls were sex- and age-matched, as indicated by the originators of the data sets [17,18]. Data sets were corrected for batch effect using the ComBat algorithm. Cochran’s Q test was used to evaluate the homogeneity of the data sets.

### 2.2. In Silico Pharmacology

The drug meta-signatures were obtained from Himmelstein et al. [24]. Briefly, the meta-signatures were generated by a meta-analysis of the Library of Integrated Network-based Cellular Signatures (LINCS) L1000 perturbation data, which includes transcriptomic profiles from >40,000 genetic and small molecule perturbations across a number of established cell lines [25,26]. Stouffer’s method for the meta-analysis of the z-scores was employed to obtain the consensus drug meta-signature [24].

Anti-signature perturbation analysis was performed using the DEGs identified for EC AD and the drug meta-signatures by nearest neighbors computation, using cosine similarity as distance metrics. A total of 1000 perturbations were used for the assessment of statistical significance. The analysis was performed using the meta-signatures of FDA-approved compounds only. Overall, 749 drugs were included in the analysis. Hierarchical clustering and similarity matrix were constructed using cosine distance on complete linkage. Analysis has been performed using the Morpheus web-based application (https://software.broadinstitute.org/morpheus/, accessed on 8 February 2022). Among the predicted drugs, we identified those with blood-brain barrier (BBB) permeability by interrogating the large benchmark data set, B3DB, which includes 7807 small molecules [27].

### 2.3. Statistical Analysis

Based on the results from the Cochran’s Q test, for the meta-analysis of the selected microarray data sets, a random-effect model was used to integrate the gene expression patterns. Genes with an adjusted (Benjamini–Hochberg corrected) *p*-value (FDR, false discovery rate) <  0.05 were identified as DEGs and used for further analysis. The web-based application NetworkAnalyst was used to perform the meta-analysis (https://www.networkanalyst.ca/home.xhtml/, accessed on 20 January 2022) [28]. Functional enrichment, gene ontology, and transcription factor analysis were conducted using the web-based utility, Metascape [29]. Unless otherwise specified, an FDR < 0.05 was considered the threshold for statistical significance.

## 3. Results

### 3.1. Identification of the AD Gene Expression Profile in EC

**Figure 2:**
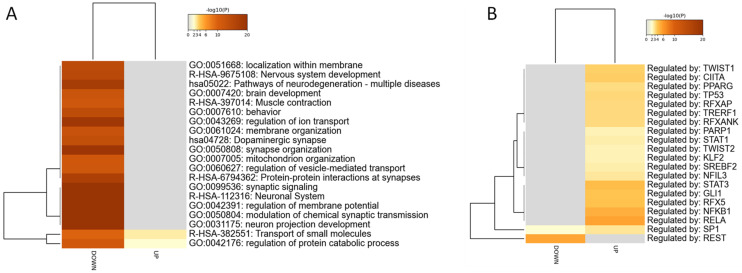
A meta-analysis of the GSE118554 and the GSE48350 data sets was performed for the identification of the differentially expressed genes (DEGs) characterizing the entorhinal cortex of AD patients and to perform a functional enrichment analysis. (**A**) Heatmap showing the most enriched terms among the upregulated and downregulated DEGs identified in the meta-analysis; (**B**) heatmap showing the putative transcription factors controlling the expression of the upregulated and downregulated DEGs identified in the meta-analysis.

Two GEO data sets were identified for the evaluation of the transcriptomic differences occurring in the EC of AD patients as compared to normal donors. A total of 36 normal control samples and 52 AD samples were used in the meta-analysis. Overall, 17,527 unique genes were included in the meta-analysis. The meta-analysis identified 1915 DEGs (Figure 2A) (a complete list of DEGs is provided in Table S1). The top 20 upregulated and top 20 downregulated DEGs are provided in Table 1 and Table 2, respectively). Among the downregulated DEGs, we found a significant enrichment of biological processes pertaining to “neuronal system” (R-HSA-112316), “synaptic signaling” (GO:0099536), “modulation of chemical synaptic transmission” (GO:0050804), “regulation of membrane potential” (GO:0042391), and “neuron projection development” (GO:0031175). In addition, ”regulation of protein catabolic process” (GO:00042176) and “transport of small molecules” (R-HSA-382551) resulted in enrichment among both the upregulated and downregulated DEGs (Figure 2A). Prediction analysis for the putative transcription factors controlling the expression of the DEGs identified RELA, NFKB1, RFX5, GLI1, and STAT3 for the upregulated DEGs and REST, and SP1 for the downregulated DEGs (Figure 2B) (the complete gene ontology analysis is presented as Table S2).

### 3.2. Prediction of Novel Chemotherapeutics for AD

**Figure 3:**
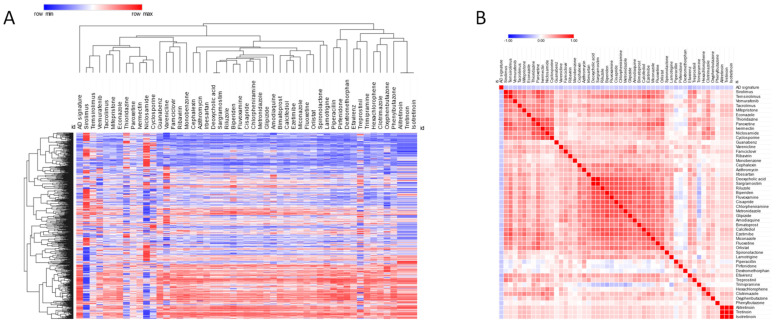
For the prediction of drugs potentially repurposable for AD, the cosine distance was calculated between the AD signature, identified from the meta-analysis of the GSE118554 and the GSE48350 data sets, and the meta-signatures of FDA-approved drugs derived from the LINCS L1000 database; (**A**) hierarchical clustering for the top 50 predicted drugs; (**B**) similarity matrix for the top 50 predicted drugs.

Anti-signature perturbation analysis was performed using the DEGs identified in AD EC and the meta-signature of drugs from the L1000 database. Overall, 170 drugs were found to have a significant anti-similarity with respect to the AD meta-signature. The top 50 drugs identified in our analysis are presented in Figure 3A,B and Table 3. Among them, the top three drugs were: efavirenz, an anti-retroviral drug; tacrolimus, a calcineurin inhibitor; and sirolimus, an mTOR inhibitor (Figure 3A,B and in Table 3) (the complete results of the drug prediction is provided as Table S3). Among the predicted drugs, we sought to identify those with BBB permeability by making use of the B3DB database [27]. Overall, 53 BBB-permeable drugs currently used in the clinical setting resulted in having a significantly anti-correlated signature with respect to the AD-related EC transcriptome. The top three drugs were: varenicline, a partial agonist for the α4β2 nicotinic acetylcholine receptor (nAChR) used for the treatment of nicotine addiction; piperacillin, a β-lactam antibiotic; and riluzole, an anti-glutamatergic drug used to treat amyotrophic lateral sclerosis (see Table 4 for the complete list of drugs).

## 4. Discussion

It is well established that dysfunction of EC is associated with the cognitive impairment occurring early in the progression of AD. Several factors seem to be implicated in the vulnerability of EC in AD, including the dysregulation of brain-derived neurotrophic factor and the ARMS/Kidins220 scaffold protein; the activation of stress-related kinases and neuroinflammatory processes; and the downregulation of neurotransmitter receptors, including N-methyl-D-aspartate (NMDA) subtype 1 glutamate receptor, muscarinic acetylcholine receptor 1, and γ-Aminobutyric acid type A (GABAA) receptor delta (reviewed in the work of [13]).

In the present study, we have employed a meta-analysis of currently available transcriptomic data sets to characterize the molecular pathways that are disrupted in the EC of patients suffering from AD. Several studies have explored the transcriptome of human brain samples in order to characterize the molecular mechanisms underlying the pathogenesis of AD [14,15,16,17,18]. The use of meta-analysis of gene expression profiles improves the power of the results and their reliability. This is particularly important as it is often difficult to compare the results from the previous studies because of a lack of concordance among them. These differences could be dependent on the different inclusion criteria for the samples and the modeling approaches used by the respective authors. For instance, while Berchtold et al. focused only on 340 synaptic genes [18], Patel et al. described 1690 DEGs between the control and AD group [17], and Miyashita et al. mostly focused on the genes with altered expression associated with neurofibrillary tangles expansion [16].

Among the transcription factors putatively involved in regulating the EC DEGs in AD patients, we found the involvement of SP1 and REST. SP1 controls amyloid precursor protein (APP) and tau expression. In addition, BACE1 has been shown to be induced by SP1 and, accordingly, it is downregulated in Sp1 knockout mice [30]. On the other hand, REST is a gene-silencing transcription factor, widely expressed during embryogenesis, which controls neuron-specific genes involved in synaptogenesis, synaptic plasticity, and neuronal remodeling (reviewed in the work of [31]). An increasing body of data indicates that dysregulation of REST is implicated in neurodegenerative diseases, including Huntington’s disease and AD. In a normal aging brain, a significant increase in REST expression is found in the nuclei of neurons of the prefrontal cortex and the hippocampus, which cannot be observed in subjects with mild or severe cognitive impairment. Moreover, REST is also reduced in frontotemporal and Lewy bodies dementia (reviewed in the work of [31]).

To date, only a few limited options are available for the treatment of AD. Mostly, these drugs are symptomatic and have a limited effect in improving cognitive function and/or slowing down the progression of the disease. Recently, the first disease-modifying agent, aducanumab, a human monoclonal antibody that selectively targets aggregated amyloid β, has been approved by the FDA [32].

Drug repurposing represents an effective strategy to accelerate the search for more effective therapeutic treatments for AD patients [33,34]. The use of drugs already approved for different indications may expedite the design of clinical trials in the AD setting, as the pharmacokinetic and pharmacodynamic properties, as well as the toxicity and the therapeutic ranges of these drugs, are already known [33,34].

In the current study, we have shortlisted potential repurposable drugs, by means of an in silico approach, based on the anti-similarity between drugs and the AD-related EC transcriptional profile [35,36,37,38,39,40].

Among the predicted drugs, the most significant was efavirenz, an anti-retroviral medication used for the treatment of HIV infection. Efavirenz is known to activate the cytochrome P450 46A1 (CYP46A1), a CNS-specific enzyme that converts cholesterol to 24-hydroxycholesterol and controls the turnover of the cholesterol in the brain. A previous study has shown that efavirenz treatment is associated with behavioral improvements in a model of AD, the 5XFAD mouse, without determining significant changes in amyloid plaque load but reducing microglia activation [41]. Interestingly, a two-center, placebo-controlled, blinded clinical trial to evaluate the safety and tolerability of efavirenz in clinically stable subjects with MCI/early dementia due to AD is currently ongoing (ClinicalTrials.gov Identifier: NCT03706885).

In addition, tacrolimus and cyclosporine have been identified in our analysis as drugs potentially repurposable for AD. These drugs inhibit calcineurin, an important phosphatase known to modulate synaptic activity and memory formation. It was previously shown that calcineurin mediates the neurotoxic effects of Aβ oligomers, and elevated calcineurin levels have also been found in the CNS of AD patients [42]. Accordingly, a large retrospective study reported that the prevalence of dementia and AD in organ-transplanted patients treated with calcineurin inhibitors is significantly lower as compared to national data from the general population [42].

It is also worth mentioning that sirolimus and temsirolimus were among the top 50 predicted drugs. These drugs are targeted inhibitors of mTOR. In the CNS, mTOR and its signaling pathway control synaptic plasticity, memory, and neuronal recovery. mTOR hyperactivity has been found in AD brains, and it may represent one of the key events contributing to the formation of toxic aggregates during AD pathology [43]. Accordingly, inhibition of mTOR by sirolimus reduces the cognitive deficits and the amyloid-β levels in a mouse model of Alzheimer’s disease, the PDAPP transgenic mouse model [44].

However, it is worth mentioning that many of the currently approved drugs are not permeable to BBB, which may limit the translatability of the findings provided here. Therefore, by interrogating the D3DB database [27], we have identified the drugs, among the predicted ones, that are presumably able to cross the BBB. Among the top three drugs, we found varenicline and riluzole.

Varenicline is a selective α4β2 nAChR partial agonist in use as a smoking cessation aid. Previous reports showed that the cognitive deficits occurring in AD patients are associated with a marked reduction in α4β2 nAChRs [45], and that the expression levels of α4 and β2 nAChR subunits significantly correlate with the cognitive test scores [46]. Accordingly, in preclinical models, cognitive improvements were observed upon varenicline administration [47], and in a clinical trial on 14 smokers with schizophrenia, varenicline was associated with better cognitive test scores for verbal learning and memory [48]. However, an initial evaluation of varenicline in AD patients did not lead to any significant effect [49]. It remains to be ascertained whether different varenicline doses, administration regimens, or duration of treatment could be effective in improving the symptoms of AD.

Riluzole, a glutamate modulator used as a treatment in amyotrophic lateral sclerosis, has been previously associated with the prevention of senile cognitive impairment [50], and in preclinical rodent models of AD, riluzole has been shown to hinder cognitive decline [50,51]. Our findings, along with the available preclinical data, open up new therapeutic opportunities for the development of approaches to treat AD patients by regulating glutamate excitotoxicity affecting vulnerable brain circuits.

On a final note, we should mention that there are some drawbacks to our study. A limitation of our method is represented by the fact that our approach does not account for post-transcriptional modifications, which may change the final phenotype. The clinical characteristics of the patients included in the datasets analyzed here are not available; hence, covariate correction was not feasible for the present meta-analysis. Moreover, no transcriptional data are currently available on the initial EC aberrations that could be observed in patients suffering from MCI. The better discrimination between the early and late events occurring in the EC from patients suffering from incipient AD would, in the future, be exploited to find more effective drugs aimed at preserving cognition and preventing the progression to AD. Finally, the drug meta-signatures that we have used come from in vitro data generated from established cell lines that do not mimic the central nervous system cellular populations and the effects of their interactions. It is also likely that many of the drugs identified in our analysis may have adverse or off-target effects. Indeed, despite our in silico approach is aimed at finding potential repurposable drugs to be used in AD, there are several shortcomings to acknowledge. First, the efficacy of a drug is determined by several factors besides the simple match of transcriptomic profiles. In addition, drugs need to reach the appropriate organ at a therapeutic concentration to have a clinically evident effect, and the route, as well as the schedule of treatment, needs to be properly determined and adapted to each patient. In doing so, it is possible to reduce the side effects while maximizing the drug efficacy. On the other hand, it should be noted that, in the present paper, we have purposely chosen drugs that are currently in use in the clinical setting. These drugs have already been characterized in terms of their pharmacokinetics, and the frequency of adverse effects is well known. Hence, we believe that our study sets the basis for future investigations on the pathogenetic processes occurring in AD and proposes the repurposing of currently used drugs for the treatment of AD patients.

## Figures and Tables

**Table 1:** Top 20 upregulated genes in AD entorhinal cortex.

EntrezID	Name	GSE48350_FC	GSE118553_FC	GSE48350_Adj Pval	GSE118553_Adj Pval	CombinedES	Adj Pval
285268	ZNF621	2.4746	0.92173	1.34 × 10^−8^	3.23 × 10^−8^	2.2507	6.12 × 10^−5^
121260	SLC15A4	0.23632	0.46129	0.15057	1.87 × 10^−9^	1.5736	0.038196
79819	WDR78	0.26701	0.43115	0.16414	6.5 × 10^−8^	1.4171	0.013998
91947	ARRDC4	1.0171	1.4368	0.17458	2.45 × 10^−8^	1.4171	0.042945
51574	LARP7	0.20582	0.34122	0.17458	7.05 × 10^−8^	1.3661	0.035172
677	ZFP36L1	0.5541	0.89219	0.17458	5.46 × 10^−8^	1.3635	0.045701
1842	ECM2	1.3153	1.3997	0.16414	3.42 × 10^−7^	1.3563	0.004831
25937	WWTR1	0.95877	1.2911	0.17458	1.78 × 10^−7^	1.3539	0.015527
79887	PLBD1	0.5105	0.59817	0.11938	1.64 × 10^−6^	1.3531	6.12 × 10^−5^
84532	ACSS1	0.32735	0.45851	0.14265	1.18 × 10^−6^	1.3436	0.000416
3769	KCNJ13	0.18731	0.36619	0.17458	3.72 × 10^−7^	1.3225	0.011159
1903	S1PR3	0.50774	0.75184	0.17458	2.56 × 10^−7^	1.3125	0.023432
828	CAPS	1.2364	1.2386	0.17458	2.52 × 10^−7^	1.2953	0.033873
3176	HNMT	0.32837	0.37213	0.081897	1.38 × 10^−5^	1.2911	1.69 × 10^−5^
6542	SLC7A2	0.88797	1.1676	0.17458	3.23 × 10^−7^	1.2669	0.043421
169792	GLIS3	0.79637	0.8679	0.16731	7.38 × 10^−6^	1.238	0.000211
137075	CLDN23	0.14904	0.33434	0.17458	3.76 × 10^−6^	1.2175	0.003207
730112	FAM166B	0.27099	0.39649	0.17458	5.21 × 10^−6^	1.2045	0.00246
83538	TTC25	0.31451	0.58902	0.17458	3.44 × 10^−6^	1.1801	0.013422
58487	CREBZF	0.15424	0.33269	0.17458	2.51 × 10^−6^	1.1742	0.024247

FC: fold change; CombinedES: combined effect size.

**Table 2:** Top 20 downpregulated genes in AD entorhinal cortex.

EntrezID	Name	GSE48350_FC	GSE118553_FC	GSE48350_Adj Pval	GSE118553_Adj Pval	CombinedES	Adj Pval
10361	NPM2	−0.3746	−0.67012	0.1148	1.12 × 10^−8^	−1.5404	0.009671
2596	GAP43	−0.61551	−0.91532	0.17458	1.28 × 10^−8^	−1.4592	0.039307
246176	GAS2L2	−0.22421	−0.24684	0.002432	3.17 × 10^−5^	−1.443	1.21 × 10^−6^
1917	EEF1A2	−0.45676	−0.73972	0.17458	3.71 × 10^−8^	−1.4062	0.032214
22859	ADGRL1	−0.34859	−0.52543	0.15557	2.14 × 10^−7^	−1.389	0.004429
10423	CDIPT	−0.19717	−0.29224	0.15057	6.77 × 10^−7^	−1.3519	0.001392
4004	LMO1	−0.68291	−0.51695	0.004138	0.000147	−1.3449	6.58 × 10^−6^
534	ATP6V1G2	−0.4706	−0.6834	0.17458	4.98 × 10^−7^	−1.2865	0.017316
51686	OAZ3	−0.56799	−0.40996	0.004184	0.000515	−1.2743	1.81 × 10^−5^
55643	BTBD2	−0.36764	−0.50131	0.17458	2.1 × 10^−6^	−1.2587	0.002795
9853	RUSC2	−0.21069	−0.386	0.17458	4.36 × 10^−7^	−1.2458	0.045965
27132	CPNE7	−0.43312	−0.66894	0.17458	8.82 × 10^−7^	−1.2335	0.026278
9556	ATP5MPL	−0.30239	−0.39911	0.17458	8.17 × 10^−6^	−1.2175	0.000416
80146	UXS1	−0.2609	−0.4633	0.17458	9.22 × 10^−7^	−1.2141	0.036783
55530	SVOP	−0.74564	−1.128	0.17458	2.11 × 10^−6^	−1.1977	0.0181
6835	SURF2	−0.34906	−0.28302	0.017189	0.000789	−1.1975	6.12 × 10^−5^
55294	FBXW7	−0.34269	−0.47962	0.17458	3.19 × 10^−6^	−1.1863	0.012874
226	ALDOA	−0.30882	−0.44703	0.17458	2.51 × 10^−6^	−1.182	0.019997
9143	SYNGR3	−0.52952	−0.78748	0.17458	3.18 × 10^−6^	−1.1782	0.015749
7280	TUBB2A	−0.33387	−0.52954	0.17458	2.34 × 10^−6^	−1.1746	0.026663

FC: fold change; CombinedES: combined effect size.

**Table 3:** Top 50 predicted drugs.

Drug	Cosine Similarity	*p*_Value	FDR
Efavirenz	−0.28	0	0.01
Tacrolimus	−0.25	0	0.01
Sirolimus	−0.25	0	0.01
Deoxycholic acid	−0.25	0	0.01
Sargramostim	−0.25	0	0.01
Bimatoprost	−0.24	0	0.01
Varenicline	−0.24	0	0.01
Calcifediol	−0.23	0	0.01
Piperacillin	−0.23	0	0.01
Treprostinil	−0.23	0	0.01
Spironolactone	−0.23	0	0.01
Cephalexin	−0.23	0	0.01
Irbesartan	−0.23	0	0.01
Thioridazine	−0.22	0	0.01
Riluzole	−0.22	0	0.01
Vemurafenib	−0.22	0	0.01
Azithromycin	−0.21	0	0.01
Phenylbutazone	−0.21	0	0.01
Mifepristone	−0.21	0	0.01
Temsirolimus	−0.21	0	0.01
Cisapride	−0.2	0	0.01
Guanabenz	−0.2	0	0.01
Trimipramine	−0.2	0	0.01
Hexachlorophene	−0.2	0	0.01
Glipizide	−0.2	0	0.01
Chlorpheniramine	−0.2	0	0.01
Econazole	−0.2	0	0.01
Pirfenidone	−0.2	0	0.01
Clotrimazole	−0.19	0	0.01
Monobenzone	−0.19	0	0.01
Ivermectin	−0.19	0	0.01
Biperiden	−0.19	0	0.01
Ribavirin	−0.19	0	0.01
Metronidazole	−0.19	0	0.01
Ezetimibe	−0.19	0	0.01
Niclosamide	−0.19	0	0.01
Cyclosporine	−0.18	0	0.01
Famciclovir	−0.18	0	0.01
Fluoxetine	−0.18	0	0.01
Dextromethorphan	−0.18	0	0.01
Alitretinoin	−0.18	0	0.01
Lamotrigine	−0.18	0	0.01
Amodiaquine	−0.18	0	0.01
Paroxetine	−0.18	0	0.01
Tretinoin	−0.18	0	0.01
Isotretinoin	−0.18	0	0.01
Orlistat	−0.18	0	0.01
Miconazole	−0.18	0	0.01
Oxyphenbutazone	−0.18	0	0.01
Fluvoxamine	−0.18	0.01	0.02

**Table 4:** Predicted drugs known to pass the blood-brain barrier.

Drug	Cosine Similarity	FDR
Varenicline	−0.24	0.01
Piperacillin	−0.23	0.01
Riluzole	−0.22	0.01
Thioridazine	−0.22	0.01
Phenylbutazone	−0.21	0.01
Temsirolimus	−0.21	0.01
Chlorpheniramine	−0.2	0.01
Cisapride	−0.2	0.01
Trimipramine	−0.2	0.01
Biperiden	−0.19	0.01
Ivermectin	−0.19	0.01
Metronidazole	−0.19	0.01
Ribavirin	−0.19	0.01
Dextromethorphan	−0.18	0.01
Fluoxetine	−0.18	0.01
Fluvoxamine	−0.18	0.02
Lamotrigine	−0.18	0.01
Paroxetine	−0.18	0.01
Tretinoin	−0.18	0.01
Chlorprothixene	−0.17	0.01
Clemastine	−0.17	0.01
Methocarbamol	−0.17	0.01
Sunitinib	−0.17	0.01
Tamoxifen	−0.17	0.01
Thiethylperazine	−0.17	0.01
Granisetron	−0.16	0.01
Loperamide	−0.16	0.01
Praziquantel	−0.16	0.01
Diphenylpyraline	−0.15	0.01
Droperidol	−0.15	0.01
Pemoline	−0.15	0.01
Saquinavir	−0.15	0.04
Sertraline	−0.15	0.01
Atomoxetine	−0.14	0.03
Cytarabine	−0.14	0.01
Gefitinib	−0.14	0.01
Sulfamethoxazole	−0.14	0.02
Pramipexole	−0.13	0.01
Naloxone	−0.12	0.01
Nortriptyline	−0.12	0.04
Pentobarbital	−0.12	0.02
Metaxalone	−0.11	0.02
Nalbuphine	−0.11	0.03
Rivastigmine	−0.11	0.01
Terbinafine	−0.11	0.02
Cyproheptadine	−0.1	0.01
Metoclopramide	−0.1	0.01
Naltrexone	−0.1	0.03
Prednicarbate	−0.1	0.02
Desoximetasone	−0.09	0.03
Ethotoin	−0.08	0.03
Zonisamide	−0.08	0.02
Flucytosine	−0.06	0.04

## Data Availability

The data presented here are a third-party reanalysis of the publicly available data sets, GSE118553 and GSE48350 data sets, which have been obtained from the Gene Expression Omnibus (GEO) database (https://www.ncbi.nlm.nih.gov/gds, accessed on 10 January 2022).

## Supplementary Materials

The following supporting information can be downloaded at: https://www.mdpi.com/article/10.3390/genes13040703/s1, Table S1: Differentially Expressed Genes in in AD entorhinal cortex; Table S2: Gene Ontology analysis of Differentially Expressed Genes in in AD entorhinal cortex; Table S3: Predicted drugs for AD.
